# Nanoparticles for Control of Biofilms of *Acinetobacter* Species

**DOI:** 10.3390/ma9050383

**Published:** 2016-05-18

**Authors:** Richa Singh, Shradhda Nadhe, Sweety Wadhwani, Utkarsha Shedbalkar, Balu Ananda Chopade

**Affiliations:** 1 Department of Microbiology, Savitribai Phule Pune University, Pune 411007, India; richasngh316@gmail.com (R.S.); nshraddha23@gmail.com (S.N.); sweety.wadhwani123@gmail.com (S.W.); 2 Department of Biochemistry, The Institute of Science, Mumbai 400032, India; utkarsha.shedbalkar@gmail.com; 3 Dr. Babasaheb Ambedkar Marathwada University, Aurangabad 431004, India

**Keywords:** *Acinetobacter*, biofilm, drug resistance, nanoparticles, phage, anti-biofilm agent

## Abstract

Biofilms are the cause of 80% of microbial infections. *Acinetobacter* species have emerged as multi- and pan-drug-resistant bacteria and pose a great threat to human health. These act as nosocomial pathogens and form excellent biofilms, both on biotic and abiotic surfaces, leading to severe infections and diseases. Various methods have been developed for treatment and control of *Acinetobacter* biofilm including photodynamic therapy, radioimmunotherapy, prophylactic vaccines and antimicrobial peptides. Nanotechnology, in the present scenario, offers a promising alternative. Nanomaterials possess unique properties, and multiple bactericidal mechanisms render them more effective than conventional drugs. This review intends to provide an overview of *Acinetobacter* biofilm and the significant role of various nanoparticles as anti-biofouling agents, surface-coating materials and drug-delivery vehicles for biofilm control and treatment of *Acinetobacter* infections.

## 1. Introduction

A biofilm is a community of single or mixed bacterial cells adhered to abiotic or biotic surfaces [1]. The biofilm is arranged in a tertiary structure where the bacteria are in intimate contact with each other and encased in a matrix of extracellular polymeric substances (EPS), which can comprise exopolysaccharides, nucleic acids, proteins and other macromolecules [2,3]. The main reasons behind bacterial biofilm formation are: (a) normal mode of growth for some species; (b) protection from adverse host environment; (c) preferential colonization in nutrient-rich conditions; and (d) co-operative benefits as a part of community [4,5]. Ubiquitous in nature, biofilms are found on rocks and pebbles in rivers, surfaces of stagnant water, showers, sewage and drinking-water pipes, marine engineering systems, ship hulls, *etc.* [6,7]. However, microbial colonization on living tissues, such as heart valves, tooth enamel, lung and middle ear, wounds, medical devices and tissue engineering-related products [5] is a matter of great concern for human health. These medical biofilms are responsible for 65% to 80% of clinical infections, which may lead to morbidity and mortality [8,9]. Bacterial cells present in these biofilms express phenotypes, different from planktonic counterparts, and exhibit higher resistance to conventional drugs, ultraviolet light, desiccation, extreme pH and host’s immune defense system [10,11,12,13]. Such biofilms have been reported in both Gram-positive and Gram-negative bacteria, including *Acinetobacter baumannii*, *Pseudomonas aeruginosa*, *Xanthomonas campestris*, *Staphylococcus aureus*, *Staphylococcus epidermidis*, *etc.* [14,15,16,17,18,19,20].

### 1.1. Acinetobacter: A Nosocomial Biofilm-Producing Pathogen

The genus *Acinetobacter* consists of 34 species, which are non-motile, aerobic and Gram-negative coccobacilli [21]. The bacteria are widely distributed in soil, activated sludge, water, food and human skin [22,23,24,25,26,27,28,29]. The bacteria can survive under highly desiccated conditions on abiotic surfaces for a long time [30,31]. In recent years, they have emerged as the most important nosocomial pathogens implicated in a variety of nosocomial infections, such as urinary and respiratory tract infections, skin and soft-tissue infections, bloodstream infections and secondary meningitis [32,33,34,35]. The treatment of *Acinetobacter* infections is becoming a challenge since these species are rapidly developing resistance to commonly used traditional antibiotics. *A. baumannii* has been listed as one of the ESKAPE pathogens (*Enterococcus faecium*, *Staphylococcus aureus*, *Klebsiella pneumoniae*, *Acinetobacter baumannii*, *Pseudomonas aeruginosa*, and *Enterobacter* species) owing to the ability to escape the biocidal activity of antibiotics [30]. They have also evolved as one of the most antibiotic and metal-resistant microorganisms [24,31]. The terms like multi-drug resistant (MDR), extensively-drug resistant (XDR) and pan-drug resistant (PDR) are used to describe the level of antibiotic insusceptibility in *Acinetobacter* spp. MDR *Acinetobacter* spp. are resistant to at least three classes of antibiotics: all penicillins and cephalosporins, aminoglycosides and fluroquinolones. XDR species are MDR plus carbapenem-resistant, whereas PDR species exhibit resistance to antimicrobials mentioned above along with polymyxins and tigecycline [36,37,38].

**Figure 1:**
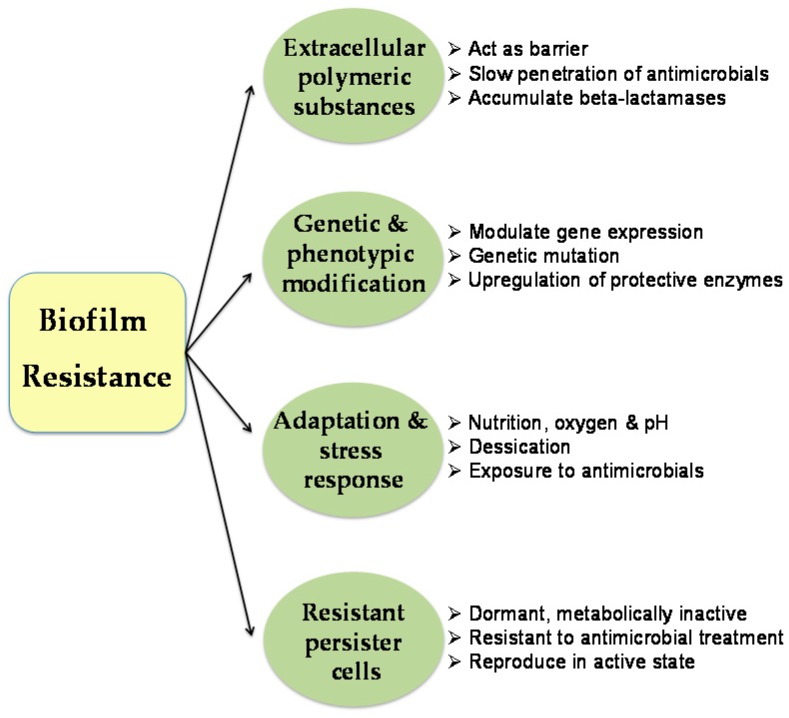
Mechanisms and factors involved in conferring drug resistance in pathogenic biofilms.

The problem is aggravated due to their colonization and biofilm-forming capacity on medical devices, such as implants, cardiac valves, artificial joints, catheters, *etc.* [32,39]. *Acinetobacter* biofilms have also been associated with hospital-acquired infections, chronic non-healing injury-and burn-wound infections, ulcers and battle casualties among military personnel [40,41]. Biofilms cause severe illness and diseases in immuno-compromised patients, especially in case of urinary and respiratory tract infections, ocular infection, otitis media, endocarditis, pneumonia, septicemia, bacteremia, and necrotizing fasciitis, *etc.* [29,42,43,44]. There is a correlation between antibiotic resistance and the ability of *Acinetobacter* to adhere to the clinically relevant surfaces, such as polystyrene and human epithelial cells [45]. Such pathogenic biofilms are heterogeneous and express up to 1000-fold drug resistance, making them difficult to eradicate [46,47]. Resistance exhibited by *Acinetobacter* biofilms can be natural, genetically acquired or adaptive to survive in that environment [3,48,49]. Although the mechanism underlying biofilm resistance is still not completely understood, it may involve the combination of factors shown in Figure 1.

### 1.2. Treatment Therapies for Control of Acinetobacter Biofilm

Molecular mechanism of biofilm formation in *Acinetobacter* needs to be understood to formulate anti-biofouling therapies. The common factors influencing biofilm formation are type of surface, nutrient availability, bacterial surface components like EPS, bacterial appendages including pili and flagella, quorum-sensing communication and extracellular organic secretions [50]. EPS of Gram-negative bacteria is anionic in nature due to uronic acids and ketal-linked pyruvates [51]. In such EPS, divalent cations, such as calcium and magnesium, facilitate crosslinking between polymeric polysaccharide strands, thereby increasing viscosity and binding forces in biofilm [51]. Quorum-sensing molecules (*N*-acyl-L-homoserine lactones, 4-quinolines) are involved in cell density-dependent intercellular communications and regulation of expression of virulence genes for exoenzymes, EPS and stress resistance [52]. Expression of genes, such as *blaPER-1* and *algC*, adhesion proteins and extracellular DNA is critical for cell adhesion, colonization and formation of biofilms [16,45,53,54,55]. Moreover, biofilm-specific housekeeping, transporter and regulatory proteins [39] can be the ideal targets for developing novel artillery to eradicate colonization and overcome biofilm resistance. Additionally, environmental and physiological factors (nutrient and oxygen availability, concentration of D-amino acids, iron, nitric oxide concentration), cell-cell communication signals (diffusible fatty acids, auto-inducing peptides) and intracellular messengers (c-di-GMP, cAMP) are a few of the molecular triggers, involved in the induction of transition from sessile phenotype to free dispersal phenotype, which can be activated to degrade the biofilms [3,20,56,57,58,59,60].

Prophylactic vaccines, antimicrobial peptides, photodynamic therapy and radioimmunotherapy are control measures employed to prevent and eradicate *Acinetobacter* biofilms [61,62,63]. Vaccination with *A. baumannii* biofilm-associated protein (Bap) and outer membrane porin (OmpA) enhances antigen-specific titers and reduces bacterial loads in intraperitoneal infection model [63,64]. Passive immunization with antibodies against membrane polysaccharides and outer membrane transporter has also been shown to elicit *in vitro* opsonophagocytolysis of *A. baumannii* [65,66]. Several peptides and their analogs, such as brevinin-2-related peptide, cationic alpha-helical skin-derived peptides and alyteserin-2a, showed excellent potency as membrane and cell disruptors against MDR and PDR strains of *Acinetobacter* [67,68,69]. Synthetic peptides and analogs can also be designed to develop novel bactericidal agents; however, they have a short life and are prone to proteolytic degradation *in vivo*.

Photodynamic therapy (PDT) is based on generation of reactive oxygen species (ROS), through photoreactive dyes, which react with target cells to damage the DNA or cellular membranes and organelles [70,71]. Owing to DNA repair machinery, major bactericidal effect of PDT is exerted due to the destruction of structural and transporter proteins and leakage of cellular contents [70]. The limitations of this therapy are restricted topical application and damage to host cells by ROS [71]. Materials, like catheters and implants, can be impregnated with antibiotics that are either embedded in the surface or designed to diffuse out [72]. This will ensure local antibiotic delivery, sustained drug release and lower systemic toxicity risks [72]. Polymers, modified dendrimer and cyclodextrin complexes and microemulsion formulations of antimicrobials are shown to be effective against bacterial and fungal biofilms [73,74,75,76].

In recent years, nanomaterials have gained significant importance in diagnosis, medicine and therapeutics as antimicrobials, antitubercular, anticancer and antidiabetic agents, antioxidants, catalysts and sensors [77,78,79,80,81,82,83,84]. Nanoparticles, with any one dimension up to 100 nm, exhibit unique physical, chemical and biological properties due to small size and possess high surface area-to-volume ratio as compared to bulk counterparts [80,81]. These characteristics render them highly effective in biological applications and make them potential candidates for development of novel nano-antibiotics. This review is intended to provide an overview of the approach on the control of *Acinetobacter* biofilms employing various types of nanoparticles, their benefits and limitations. It is also important to recognize the missing links in literature, which should be pursued further for in-depth understanding and applicability of nanoparticles.

## 2. *Acinetobacter* Biofilm Control through Nanomaterials

Both organic and inorganic nanoparticles are reported to have antibacterial and anti-biofilm potencies [14,85,86,87,88,89]. These are also used as surface-coating and drug-delivery agents [90,91] and thus offer a very promising alternative to conventional methods of biofilm control. Table 1 summarizes the various nanomaterials employed for treatment of *Acinetobacter* biofilms and infections. It is important to note that *in vivo* testing of nanoparticles has only been pursued with planktonic *Acinetobacter* [86,92,93]. Such nanoparticles, in higher concentration, may show biofilm-disruption activity.

### 2.1. Organic Nanoparticles

#### 2.1.1. Liposomes and Nanoemulsions

Liposomes are self-assembled lipid bilayers containing phospholipids, sterols, glycolipids, membrane proteins and hydrophilic polymers [101]. They resemble biological cell membranes, and can therefore act as effective drug-delivery systems. Antimicrobials can be encapsulated within the lipid bilayer (if hydrophobic), entrapped in the inner core (hydrophilic) or sequestered between the inner and outer bilayer interface (hydrophilic) of the liposome [101]. Liposomal antibiotic delivery studies have been pursued mainly in biofilm-forming *P. aeruginosa* [19,85,102]. However, in an interesting study, lipidic nanocapsules loaded with a mixture of carvacol and eugenol (phenols), cinnamaldehyde (aldehyde) and/or beta-caryophyllene (alkene) showed excellent *in vitro* antibacterial activity against *A. baumannii*. Intraperitoneal administration of this formulation resulted in increased survival in sepsis murine model [86]. Alipour *et al.* reported a decrease in bacterial count of *A. baumannii* and *A. lwoffii* when exposed to liposomal formulation of polymixin B (in 2:1 molar ratio of 1,2-dipalmitoyl-*sn*-glycero-3-phosphocholine and cholesterol). Reduction in minimum inhibitory concentration (MIC) of polymixin B was also observed [103]. Such encapsulations have the advantage of sustained and controlled release of drugs, thereby achieving effective drug-delivery and biofilm treatment with reduced cytotoxicity [101,104]. Moreover, these structures can be modified for targeted site-specific delivery.

**Figure 2:**
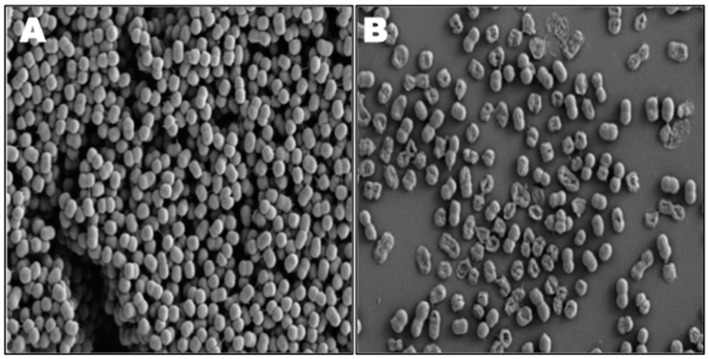
Scanning electron microscopy of MDR *A. baumannii* ATCC BAA-1605 biofilms. (**A**) Control; (**B**) Treatment with nanoemulsion of 1% cetylpyridinium chloride for 1 h (adapted from [90] with permission from © 2013 American Society for Microbiology).

Antimicrobial nanoemulsions—emulsified mixtures of detergent, oil and water with a particle size between 100–800 nm—possess a broad range of microbicidal activity against bacteria, fungi and enveloped viruses [105]. Figure 2 shows the disruption of *A. baumannii* biofilm on exposure to nanoemulsion of cetylpyridinium chloride, a quaternary ammonium salt. The nanoemulsion not only penetrates the thick biofilm matrix but also damages the bacterial cells [90]. These emulsified nanoparticles act by fusing with lipid bilayers and destabilizing the cell membrane [106]. In addition to liposomes and nanoemulsions, solid lipid nanoparticles, lipoproteins and micelles can also be used for drug delivery [107].

#### 2.1.2. Polymeric Nanoparticles

Polymers are multifunctional biomaterials that can be engineered for wide properties suitable for applications in medicine and pharmaceutical industry as drug carriers, surgical sutures, scaffolds and resorbable devices [108,109,110]. While some polymers possess antimicrobial activity due to specific functional groups, such as halogens, guanidine or quaternary nitrogen atom [111], few of the other polymers can also be loaded with antimicrobial agents. The functional groups on polymer nanoparticles can be modified or novel synthetic analogs can be designed to increase their specific activity and selectivity. Properties of biocompatible polymers can also be harnessed for *in vivo* applications. However, very few reports are available on inhibition and disruption of *Acinetobacter* biofilms through polymers [92,112]. Maleic anhydride-based amphiphillic polymers, containing amide side chains, disrupt surface established *A. baumannii* biofilms. These polymers also reduce the bacterial count in mice with chronic burn-wound infection [88]. Similar observation was seen with methacrylate polymers containing a 2-aminoimidazole subunit [112]. Chitosan nanoparticles act as adjuvant to carry outer membrane proteins of *Acinetobacter* and elicit excellent immune response in rats [92], indicating potential for developing a novel vaccine. Poly(lactic-co-glycolic acid) polymeric nanoparticles have been used for effective delivery of antibiotics to treat biofilm-forming microorganisms [113]. Nylon-3-polymers and antimicrobial polymeric hydrogels can also be employed for the control of MDR bacterial and fungal biofilms [114,115].

### 2.2. Inorganic Nanoparticles

#### 2.2.1. Silver Nanoparticles

Silver and its compounds are well known for antimicrobial properties and have been widely used in medicine and therapeutics for treatment of wounds, burns and infections. Nano-sized silver particles, however, exhibit superior antimicrobial activity against both Gram-positive and Gram-negative pathogenic bacteria, mycobacteria, fungi and yeasts [87,116,117,118]. There are many reports confirming inhibition and disruption of biofilms on exposure to silver nanoparticles (AgNPs) [119,120]. These particles have been used as disinfectant filters and surface-coating materials for implants and medical devices to prevent bacterial growth and infection [119,120,121,122]. In an interesting study, AgNPs synthesized from environmental *A. calcoaceticus* showed excellent disruption capability on preformed biofilms of clinical *A. baumannii* and *A. haemolyticus* strains isolated from hospitals [94]. Similar results were observed with AgNPs synthesized through reduction by gallic acid [91] and root extract of *Plumbago zeylanica*, a medicinal plant [15]. Nanosilver, owing to its small size, can easily penetrate the thick EPS in biofilms [94].

Synergy between AgNPs and conventional drugs offers a promising approach to control biofilm-related infections. Exposure to a combination of AgNPs with various antibiotics increases the drug susceptibility of planktonic MDR *A. baumannii* [87]. Formulation of imipenem and AgNPs not only killed planktonic cells but also eradicated their biofilm [95]. Combined killing mechanism exerted by antibiotics and AgNPs increases the susceptibility of MDR strains towards antibiotics and makes it difficult for bacteria to thrive in biofilms. Such an approach will help in combating drug resistance among *Acinetobacter* species.

#### 2.2.2. Gold Nanoparticles

Gold nanoparticles (AuNPs) provide stable, non-toxic and biocompatible alternative, which can be easily synthesized in various morphologies, such as nanospheres, nanorods, nanoshells and nanocrystals [78,123]. Since AuNPs exhibit biocompatibility, surface plasmon resonance and photothermal effect, they have found wide applications in sensors, diagnosis and cancer treatment. Although few reports describe the antibacterial activity of AuNPs [124,125], Salunke and coworkers reported poor efficacy of chemical and phytogenic AuNPs to inhibit and disrupt *A. baumannii* biofilm [15]. However, these particles are known to carry therapeutic payloads, such as antibiotics, bound to them by covalent bonding, electrostatic adsorption, encapsulation or non-covalent interactions [126,127]. These moieties are triggered through internal and external stimuli [126]. For example, vancomycin-bound AuNPs showed successful hyperthermic killing of Gram-positive and Gram-negative pathogens including PDR *A. baumannii* via near infra-red irradiation [96]. According to Cui *et al.*, AuNPs alter membrane potential, decrease intracellular ATP levels, and inhibit activity of ATP synthase and tRNA-binding subunit of ribosome [125]. Surface modification of AuNPs has also been suggested to control their inhibitory effects [128].

#### 2.2.3. Selenium Nanoparticles

Selenium nanoparticles (SeNPs) exhibit good absorption capacity, higher bioavailability and reduced cytotoxicity to have medicinal applicability [81]. However, only a single study demonstrated the anti-biofilm activity of actinobaterially synthesized SeNPs. Complete biofilm inhibition in six drug-resistant *Acinetobacter* strains was observed at 3.2 μg concentration of SeNPs in 48 h [89]. Mechanism of antibacterial action is still unknown.

#### 2.2.4. Nitric-Oxide Releasing Nanoparticles

Nitric oxide (NO) is a lipophilic, short-lived free radical with a very small size that allows it to easily diffuse across membranes and interact with both extra- and intra-cellular components [3]. NO and its derivatives cause nitrosative stress on biological membranes and DNA damage through N-nitrosation and oxidative cleavage; they also interact with thiol-containing protein via S-nitrosation and provoke lipid peroxidation leading to membrane disruption [129]. Exposure to low doses of NO restores biofilm sensitivity towards a variety of antimicrobial agents, thereby increasing their efficacy in dispersing bacteria [3]. Topical application of NO-releasing nanoparticles (~10 nm) reduces the bacterial load, inflammation and collagen degradation, as well as modulates cytokine response with a substantial decrease in healing time in *A. baumannii* wound infection [93]. Since their formulation can only be applied on the skin surface, the use is prevented in common *Acinetobacter* infections, bacteremia and pneumonia [71]; however, they make an attractive alternative for environmental biocontrol and treatment of wounds, burns and other skin-related infections.

#### 2.2.5. Multi-Metallic Nanoparticles

Use of bi- and tri-metallic nanoparticles is a great approach whereby, instead of a single metal, properties of two or more metals can be exploited. Such nanoparticles exhibit enhanced medicinal and therapeutic efficacy and are required in low concentrations to achieve a similar bactericidal effect as that with mono-metallic ones. Phytogenic silver-gold bimetallic nanoparticles from root extract of *P. zeylanica* showed significant inhibition and disruption of preformed *Acinetobacter* biofilm [15]. In another report, gold-silver core-shell nanoparticles from medicinal plant *Dioscorea bulbifera* inhibit biofilm formation among both Gram-positive and Gram-negative bacteria, including *A. baumannii* [14]. The bactericidal effect from these nanoparticles is due to cell-wall damage causing efflux of cellular materials, which may be attributed to the presence of silver [14,15]. Once the pores are made in the cell wall, silver and gold interact with cellular components and DNA to cause more destruction to bacteria. Although no report describes the efficacy of tri-metallic nanoparticles in control of *Acinetobacter* biofilm, the study of Mahmoodi and Serpooshan confirmed that chemically prepared tri-metallic SPIONs, consisting of gold and silver shells onto iron core, have profound anti-biofilm potency against *S. aureus* and *S. epidermidis* [18].

### 2.3. Nanoconjugates, Nanoalloys and Nanocomposites

**Figure 3:**
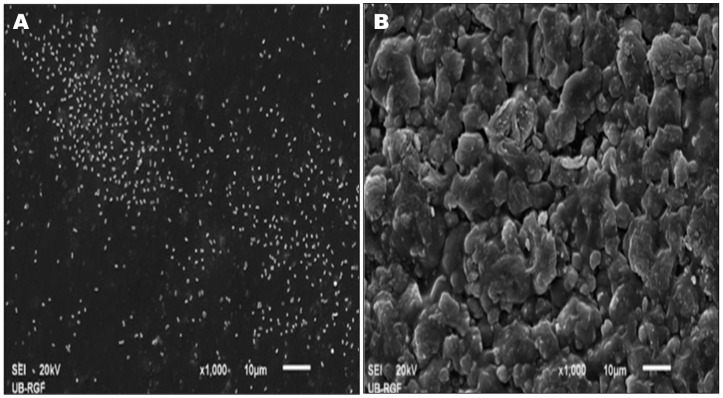
Effect of D-tyrosine coating on composite. (**A**) *A. baumannii* cells without biofilm formation on uncoated composite; (**B**) Absence of bacterial cells on composite coated with D-tyrosine. One side of the composite surface was (**A**) shiny while other side was (**B**) coarse (obtained from [98], with permission from © 2014 Taylor & Francis Ltd).

Thus far, nanoconjugates and nanoalloys have not been employed to inhibit *Acinetobacter* colonization. However, profound reduction in *A. baumannii* has been reported on treatment with nanocomposites, synthesized by copper-based nanostructured coating on natural cellulose substrate. Relatively low efficacy was observed with similar silver-coated nanocomposite [97]. Studies have confirmed antibacterial and anti-biofouling activity of micron-sized alloys and composites [98,130], which is dependent on the constituents, type of surface and coating materials. For example, surface modification of titanium implants through doping with silver and/or gallium enhances antibacterial effectiveness against MDR *A. baumannii* [130]. Figure 3 depicts 100% killing of *A. baumannii* on a composite containing silver-exchanged natural zeolite and poly(vinyl chloride), coated with D-tyrosine. Uncoated composite inhibits the biofilm formation on the surface with only 70% reduction in bacterial load [98]. D-tyrosine gets incorporated into the peptidoglycan layer of the bacterial cell wall and replaces D-alanine, thereby disrupting the cell connection with the biofilm matrix [58,131,132].

### 2.4. Bacteriophages as Living Nanobullets

Lytic phage therapy employs viruses that infect and lyse the bacterial cells. Lytic phages specific to clinical and MDR *Acinetobacter* strains have been isolated from sewage, marine water, patient sputum, *etc.* [71]. Two phages, AB7-IBB1 and AB7-IBB2, specific to *A. baumannii* AIIMS 7 reported from our laboratory have been shown to act as anti-biofilm agents inhibiting biofilm formation and eradicating up to 75% preformed biofilm [99,100]. In another study, a cocktail of phages was observed to lyse 113 of 127 *A. baumannii* strains [133], indicating their utility in hospital and environmental biocontrol. However, host-range specificity and *in vivo* studies need further investigation.

## 3. Resistance towards Nanoparticles

Conventional drugs are losing their functional value due to the rapidly developing drug resistance in microorganisms. This insusceptibility prompted researchers to exploit nanoparticles as an alternative approach to deal with aggressive pathogens like *Acinetobacter*. Multiple mechanisms have been reported to explain the bactericidal action of nanoparticles: they can penetrate EPS, disrupt cellular morphology, inactivate vital enzymes and proteins, denature proteins, generate ROS, inhibit DNA replication and prevent ribosome interactions [80,134,135,136]. Such multi-mode bactericidal action of nanoparticles is beneficial since bacteria would have to develop a number of mutations simultaneously to survive [80]. However, this raises concerns on the specificity of nanoparticles to kill a particular pathogen. Unlike traditional antibiotics, inherited resistance towards organic and inorganic nanoparticles has not been observed in bacteria. However, a recent study suggested that bacteria could evolve to acquire resistance through genetic mutations on continuous treatment with AgNPs for 225 generations [137]. Hence, care should be taken to avoid unintentional and unnecessary exposure of microorganisms towards these nanoparticles.

## 4. Future Prospects

*Acinetobacter* spp. are emerging as biofilm-producing, multi-drug resistant (MDR) nosocomial pathogens due to which antibiotics and natural phytogenic extracts are rendered ineffective in their control. Although nanomaterials have shown a great potential to curb *Acinetobacter* threats, in-depth studies are required to develop a potent and permanent solution. First and foremost, nanoparticles effective against planktonic *Acinetobacter* should be investigated further for their biofilm-disruption activity. Among organic nanoparticles, solid lipid nanoparticles, nanoemulsions, lipoproteins and micelles can also be used for targeted drug-delivery systems. In spite of a large number of polymers, very few polymeric nanoparticles have been investigated. Recently, carbon nanotubes, graphenes and fullerenes have gained medicinal significance and they may have potential to prevent *Acinetobacter* biofilms and infections. It is apparent from the earlier sections of this review that organic nanoparticles are effective mainly as drug-delivery vehicles owing to their biocompatible nature and ease of surface modification. However, only a single study demonstrates the increased survival rate in a sepsis murine model [86]. Although *Acinetobacter* species are well known to cause various biofilm-related internal infections, there are no reports on *in vivo* testing of these nanoparticles on established biofilms of *Acinetobacter*.

There are a number of reports on bactericidal and biofilm-disruption activity of metal and metal oxide nanoparticles of copper, titanium, titanium oxide, zinc, zinc oxide and iron. In addition to these, therapies based on gallium, magnesium, calcium and aluminum-derived nanoparticles can also be used. Inorganic nanoparticles, though they exhibit excellent bactericidal properties, always encounter biocompatibility, cytotoxicity and genotoxicity concerns. For these reasons, many researchers do not recommend their *in vivo* applications. On the contrary, reports are available supporting the non-toxic nature of metal nanoparticles [79,97]. Therefore, there is a need for further investigation to have a clear understanding of the toxicity aspect of these nanoparticles. The possibility of a balanced dosage of nanoparticles to achieve effective treatment without side effects cannot be ruled out.

In addition, a combination of metals and/or polymers in the form of nanoconjugates, nanoalloys and nanocomposites can be developed to enhance their biocompatibility and biofilm-disruption activity. Synergistic action of nanoparticles in combination with various antibiotics resulted in excellent inhibition of bacteria in planktonic stage. These combinations not only render ineffective antibiotics to kill bacteria efficiently, but also reduce their minimum inhibitory concentration (MIC). This synergistic approach will certainly reduce the therapeutic dose to cure the bacterial infections, thereby reducing the toxicity risks. Furthermore, formulations of nanoparticles should be developed for topical application. These will prove to be very helpful in treatment of burns and injuries, healing wounds and prevention of *Acinetobacter* infections. Application of nanomaterials should also be investigated as coating agents on surfaces of medical devices, implants, contact lenses and industrial machineries. Source, surface, composition and morphology-dependent action of nanoparticles should be evaluated. Although phage therapy is promising for hospital and environmental biocontrol, *in vivo* applications require further investigations. Host specificity of phages can be overcome by exposure to a phage cocktail.

Along with the factors influencing biofilm formation, physiological adaptation to stress, slower metabolism and increased expression of biofilm-specific traits—such as accumulation of β-lactamases, periplasmic antibiotic-binding polysaccharides, type IV pili or upregulation of enzymes to protect against endogenous oxidative stress, outer membrane proteins and porin channels—have been suggested to play a significant role in biofilms. Nanoparticles have been shown to penetrate the extracellular polymeric substances (EPS) of biofilms causing its disruption. However, detailed studies are required on molecular and genetic expression in biofilm, in response to treatment with nanoparticles, to elucidate the bactericidal and biofilm-disrupting mechanisms of nanoparticles. This will certainly aid in combating MDR biofilms and development of novel nano-formulations.

## Figures and Tables

**Table 1:** Nanomaterials in control of *Acinetobacter* biofilms and infections.

NPs	Composition and Surface Property	Size (nm)	*Acinetobacter* Strain	Applied Dosage of NPs	Remarks	Ref.
*Lipid-based NPs*						
Lipidic nanocapsules	(1) carvacol, eugenol and cinnamaldehyde (0.96% w/w) (2) carvacol (0.34% w/w), eugenol (1.83% w/w), cinnamaldehyde (0.39% w/w) and β-caryophyllene (0.32% w/w)	85–95 62–70	*A. baumannii*	40 mg/kg	Increased survival in sepsis murine model	[86]
Nanoemulsion of CPC	CPC (1% w/v), triton X-100 (10% v/v) and soyabean oil (25% v/v)	213.9	*A. baumannii* ATCC BAA-1605	~5–25 μg/mL CPC	Loss in metabolic activity; complete biofilm disruption	[90]
*Polymer-based NPs*						
Chitosan NPs	OMP loaded on NPs	-	*A. baumannii*	533 + 170 μg/mL (OMP + chitosan) 1st and 3rd week: 0.5 mL; 5th week: 1 mL	Modulate cytokine profile; trigger immune response; act as nano-vaccine	[92]
*Inorganic NPs*						
AgNPs		12.05	*A. baumannii* SRMC 27; *A. haemolyticus* MMC 8	2000 μg/mL	80%–92% biofilm inhibition and disruption	[94]
		21–29	*A. baumannii* ATCC BAA-1605	250–1000 mg/mL	Biofilm disruption on polycarbonate membrane; ~4-log reduction in cell load at highest concentration	[91]
	Combined with imipenem	-	*A. baumannii*	0.0003–0.8 μg/mL	Synergistic action; reduced MBIC and MBEC	[95]
		60	*A. baumannii* AIIMS 7	1024 μg/200 μL well	96%–99% biofilm inhibition; 88% eradication; change in cell morphology	[15]
AuNPs	Vancomycin bound	-	*A. baumannii*	-	Hyperthermic bactericidal action via NIR irradiation	[96]
Silver-gold bimetallic NPs		90	*A. baumannii* AIIMS 7	1024 μg/200 μL well	93%–98% biofilm inhibition; 61%–77% eradication; cell lysis	[15]
	Au (core) and Ag (shell)	13–19	*A. baumannii*	100 μg/mL	83% biofilm inhibition	[14]
SeNPs	-	100–250	*Acinetobacter* sp. (4117, 1677, 2030, 674, 2020, 1370)	1.2–3.6 μg/mL	Dose-dependent anti-biofilm activity; 75% reduction	[89]
Nitric oxide-releasing NPs	Composite matrix of TMO, PEG, chitosan and glucose with sodium nitrite	10	*A. baumannii* 0057	5 mg	Reduced wound healing time *in vivo*; reduced inflammatory response; inhibited collagen degradation; induced cytokine expression	[93]
*Nanocomposites*						
Cu^1^-based NPs in natural cellulose	Bare metal or metal oxide coating	<5	*A. baumannii*	~30 μg Cu in liquid culture	Bactericidal action without cytotoxicity	[97]
Ag^1^-based NPs in natural cellulose	Bare metal or metal oxide coating	-	*A. baumannii*	~12 μg Ag in liquid culture	Bactericidal activity; toxic to NIH 3T3 cell line	[97]
Ag-exchanged zeolite	Coated with D-tyrosine	500–1500	*A. baumannii* ST145	-	Complete bactericidal activity towards immobilized cells; 6.9-log cell reduction	[98]
*Bacteriophages*						
AB7-IBB1	*Siphoviridae* family	50 (head); 240 × 10 (tail)	*A. baumannii* AIIMS 7	MOI 10^5^ with 10^2^ CFU ^1^/well	Lyse 23 of 39 clinical isolates of *A. baumannii*; affected biofilm formation on biotic and abiotic surface; 75% eradication of biofilm	[99]
AB7-IBB2	*Podoviridae* family	35 (head); 7 (tail)	*A. baumannii* AIIMS 7	MOI 10^5^ and 10^3^ with 10^2^ and 10^4^ CFU/well, respectively	Lyse 19 of 39 clinical isolates of *A. baumannii*; affected biofilm formation on biotic and abiotic surface; 80% eradication of biofilm	[100]

^1^ NPs, nanoparticles; CPC, cetylpyridinium chloride; OMP, outer membrane protein; AgNPs; silver nanoparticles; MBIC, minimum biofilm inhibitory concentration; MBEC, minimum biofilm eradication concentration; AuNPs, gold nanoparticles; NIR, near infra-red; SeNPs, selenium nanoparticles; TMO, tetramethyl-orthosilicate; PEG, polyethylene glycol; Cu, copper; Ag, silver; MOI, multiplicity of infection; CFU, colony-forming unit; “-” not reported.
